# Mitochondrial Gene Expression Profiles and Metabolic Pathways in the Amygdala Associated with Exaggerated Fear in an Animal Model of PTSD

**DOI:** 10.3389/fneur.2014.00164

**Published:** 2014-09-23

**Authors:** He Li, Xin Li, Stanley E. Smerin, Lei Zhang, Min Jia, Guoqiang Xing, Yan A. Su, Jillian Wen, David Benedek, Robert Ursano

**Affiliations:** ^1^Department of Psychiatry, Center for the Study of Traumatic Stress, Uniformed Services University of the Health Sciences, Bethesda, MD, USA; ^2^Department of Biostatistics, Bioinformatics, and Biomathematics, Georgetown University Medical Center, Washington, DC, USA; ^3^Department of Gene and Protein Biomarkers, GenProMarkers, Rockville, MD, USA

**Keywords:** PTSD, amygdala, mitochondrial genes, stress, fear, Bcl-2, microarray

## Abstract

The metabolic mechanisms underlying the development of exaggerated fear in post-traumatic stress disorder (PTSD) are not well defined. In the present study, alteration in the expression of genes associated with mitochondrial function in the amygdala of an animal model of PTSD was determined. Amygdala tissue samples were excised from 10 non-stressed control rats and 10 stressed rats, 14 days post-stress treatment. Total RNA was isolated, cDNA was synthesized, and gene expression levels were determined using a cDNA microarray. During the development of the exaggerated fear associated with PTSD, 48 genes were found to be significantly upregulated and 37 were significantly downregulated in the amygdala complex based on stringent criteria (*p* < 0.01). Ingenuity pathway analysis revealed up- or downregulation in the amygdala complex of four signaling networks – one associated with inflammatory and apoptotic pathways, one with immune mediators and metabolism, one with transcriptional factors, and one with chromatin remodeling. Thus, informatics of a neuronal gene array allowed us to determine the expression profile of mitochondrial genes in the amygdala complex of an animal model of PTSD. The result is a further understanding of the metabolic and neuronal signaling mechanisms associated with delayed and exaggerated fear.

## Introduction

The neurobiology of exaggerated fear is important in understanding traumatic stress and post-traumatic stress disorder (PTSD). Decades of animal studies have shown that the amygdala is key in the neuronal system that orchestrates the fear memory and exaggerated fear evoked by stress. In PTSD patients exposed to an aversive visual stimulus or a fear inducing pulse of sound, fMRI shows an increased blood flow and metabolic rate in the amygdala, indicating a prolonged dysregulation of metabolism and an altered neuronal transmitter system in the amygdala circuitry (1–3). Dysregulation appears to persist throughout life, at least in the frontal cortex, as we found by analyzing a mitochondria gene array from post-mortem frontal cortex samples of PTSD patients. Genes and signaling pathways, as well as pharmacological targets, were significantly dysregulated (4).

Mitochondria are targets for stress hormones such as corticosterone (CORT) and are increasingly recognized as key components in stress-related mental disorders (5, 6). Prolonged and repetitive psychological stress can induce long-lasting neurobiological consequences. However, the exact subcellular mechanisms involved in such long-term neuronal and hormonal impairment remain elusive. The current study used a rat model of PTSD to further examine amygdala function and traumatic stress. In this study, we examined the relationship of altered amygdala mitochondrial-function genes and the molecular signaling pathways associated with a key symptom of PTSD: delayed and exaggerated fear. Amygdala tissues were collected from rats 14 days after stress, a time point at which exaggerated fear manifests. We then screened for 1500 mitochondrial-function-associated genes, including 37 mitochondrial DNA (mtDNA)-encoded genes, 1,098 nuclear DNA (nDNA)-encoded and mitochondria-focused genes, and 365 neuron-related genes (7, 8).

Up until now, the altered subcellular and metabolic molecular markers associated with delayed and exaggerated fear in the amygdala were unknown. Thus, this research was carried out in an attempt to determine whether traumatic stress alters the expression profiles of mitochondrial genes in association with the pathogenesis of delayed and exaggerated fear. A mitochondrial microarray technique was used to examine all mitochondrial-function-associated transcriptomes and to map the susceptible gene clusters in the amygdala complex associated with delayed and exaggerated fear. Our results indicate that signaling pathways, including pre-inflammatory, apoptotic, metabolic, neuronal transmitter system, and genes of G-protein-coupled receptors are up- or downregulated 14 days after the stress paradigm. The impacts of alteration in regulation of these molecular mechanisms are discussed below.

## Materials and Methods

### Animal model of PTSD

The animal model of PTSD employs restraint and tail shocks on three consecutive days. This inescapable tail-shock model (ITS) in rats mimics in many ways the pathophysiology of PTSD (9–13). In our model, stress exposure consists of a daily 2-h session of immobilization and tail-shocks for three consecutive days. The animals are restrained in Plexiglas tubes, and 40 electric shocks (2 mA, 3 s duration) are applied at varying intervals (140–180 s). Animals thereby undergo an aversive experience under conditions in which they cannot adaptively respond. We and others have verified that the ITS model induces behavioral and neurobiological alterations similar to those found in PTSD subjects (9–12). Specifically, these stressed rats exhibit (1) a delayed and exaggerated startle response appearing several days after stressor cessation which, given the compressed time scale of the rat’s life compared to a humans, corresponds to the 1–3 months delay of symptoms in PTSD patients (14, 15), (2) enhanced plasma CORT for several days, indicating a compromised hypothalamopituitary axis (HPA), and (3) retarded body weight gain after stressor cessation, indicating dysfunction of gene expression. The gene expression microarray used in this experiment, dubbed the rat mitochondrion-neuron focused microarray (rMNChip), focuses on mitochondrial and mitochondria-related nuclear genes in the rat so as to specifically address the neuronal bioenergetics hypothesized to be involved in arousal, CORT, and growth as addressed in our model of PTSD (13).

Male albino Sprague Dawley rats were used (Taconic Farms, Derwood, MD, USA), weighing 150–200 g at the time of administration of the stress protocol. Two groups of animals (*n* = 20) were studied. Twenty animals were assigned to each group based on their body weight and baseline startle response. Group 1 received the stress protocol, and Group 2 was the control. Stress exposure consisted of a daily 2-h session of restraint by immobilization in ventilated Plexiglas tubes (“inescapable”) and tail shocks for three consecutive days. Stressing was performed in the morning (between 08:00 and 12:00 hours).

Forty electric shocks (2 mA, 3 s duration; Animal Test Cage Grid Floor Shocker, Coulbourn Instruments, USA) were delivered to the tail of Group 1 rats at semi-random intervals of 150–210 s (Graphic State Notation Software, Habitest Universal Link, Coulbourn Instruments, USA). Electrode gel was applied using *Q*-tips to form a thin layer of conducting gel between the electrode and the skin of the rat’s tail. The electrode clips were adjusted and connected to the tail to ensure a good connection without affecting the blood circulation of the tail. Amygdala dissection was performed as previously described (13).

### Rat mitochondria neuronal chip

The rMNChip was designed and fabricated by GenProMarkers, Inc. (Rockville, MD, USA) using published methods (8). Briefly, the rMNChip contains 1,500 genes, including 37 mtDNA-encoded genes, 1,098 nDNA-encoded and mitochondria-focused genes, and 365 genes associated with neuronal functions, including fear response, circadian rhythms, intraneuronal signal transduction, and neurotransmitters. The oligonucleotides used were designed with the software MacVector v10.6.0 (MacVector) using full-length mRNA sequences as the templates according to published criteria. An amino-C6 modifier was added to the 5′-end of each oligonucleotide probe to enhance the binding of the DNA to glass slides and the accessibility of hybridization with target DNA. The 1,500 test genes (including 80 “housekeeping” genes as positive controls) and 36 negative controls (non-rat DNA) were printed, each in triplicates, onto the *N*-hydroxysuccinimide ester reactive group-coated glass slides (CodeLink Activated Slide, SurModics, Eden Prairie, MI, USA). DNA probes in the print buffer (50 mM sodium phosphate), at a final concentration of 20 μM of 5′-amino-C6 modified 50-mers, were printed in the Class 100 super-clean environment as described previously (7, 16, 17), using 100 μM pins and the GeneMachine OmniGrid 100 Microarrayer (Genomic Solutions, Ann Arbor, MI, USA).

### RNA purification

Total cellular RNA was purified from rat brain tissues by using a PAXgene RNA Kit (QIAGEN Inc., Valencia, CA, USA) following the manufacturer’s instructions. The amygdalas of rats from both the stress and control groups were dissected immediately after euthanasia (13).

### Microarray hybridization

One microgram of RNA per sample was used for Cy5dUTP labeling of cDNA by use of the express array detection kit (3DNA Array 900, Genisphere, Hatfield, PA, USA) following the manufacturer’s instructions. Slides were scanned using 5 μM resolution and the LOWESS method with a ScanArray Microarray Scanner (PerkinElmer). Triplicate microarray experiments were performed for each RNA sample purified from the amygdala. The background-subtracted mean values of the measured gene expression signal intensities were used for microarray data analysis. All microarray experiments were performed in the same laboratory as GenProMarkers, Inc. (8).

### Microarray database, bioinformatics, and systems biology

A gene expression database was constructed using FileMaker software (FileMaker Pro, Inc., Santa Clara, CA, USA). Database construction, data filtering, selection, exclusion and inclusion procedures, and criteria were performed as described previously (7, 8). The quantile normalization method (18) in software R version 2.15.1 (The R Foundation for Statistical Computing) was used to normalize background-subtracted mean intensities across all intra- and inter-slides of informative microarray data. Correlation and Single linkage methods were used to cluster and to make the heatmap using Cluster version 3.0 and MapleTree softwares^1^. The normalized data were also used for the calculation of means, SD, fold changes, moderated *p*-values, and false discovery rates (FDR). Gene IDs, official symbols, and official full names were updated using the NCBI database^2^. Kyoto Encyclopedia of Genes and Genomes (KEGG) pathways and Online Mendelian Inheritance in Man (OMIM) were from DAVID Bioinformatics Resources^3^.

### Statistics

The quantile normalization method (18) in software R/Biocon- ductor version 2.15.1 (The R Foundation for Statistical Computing) was used to normalize data. Means, SDs, and fold changes were calculated from triplicate spots (Amygdala complex) and triplicate experiments using XLSTAT 2006 (XLSTAT, New York, NY, USA). Differentially expressed genes were identified arbitrarily as having a greater than twofold change in the average expression of the background-subtracted mean intensity ratios of a gene between comparisons (19). The moderated *p*-values and FDR for multiple statistical testing with Benjamini and Hochberg methods (20) were calculated with the software R/Bioconductor version 2.15.1 (The R Foundation for Statistical Computing). The level of statistical significance was set at a *p*-value <0.01 with a specific FDR indicated. The error bar and plot were generated using Origin Lab version 8.5.

### Experimental paradigm (Figure 1)

**Figure 1:**
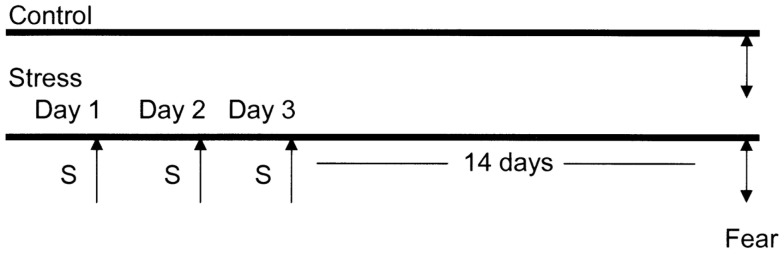
**Diagrammatic representation of the animal stress protocol**. Amygdala complex (A) from control group (*n* = 10) and stressed group (*n* = 10) were dissected 14 days after cessation of 3 days stress (S).

## Results

### Heatmap presentation following microarray data collection and analysis

The amygdala tissues were dissected from the rat brain 14 days after the cessation of the 3 days of stress. Total RNA samples were extracted from the amygdala complex of stressed rats (*n* = 10), and controls (*n* = 10) (13). Microarray experiments were triplicated using our recently developed fourth generation rat mitochondria-focused cDNA chip rMitChip3. With three spots in one cDNA microarray chip and three chips per each amygdala sample, the resulting analysis of nine data values from each single specimen greatly reduces misclassification rates (8). The measurement of expression of individual genes on the rMitChip3 (rMNChip) in one group was analyzed using 90 values (3 identical probes per microarray, 3 microarray experiments per specimen, and 10 specimens per gene), which generated reliable expression data for further pathway analysis. In this present study, the mitochondria-focused gene expression profilings in the amygdala complex 14 days after traumatic stress were analyzed using the cDNA microarray chip containing 1500 genes associated with mitochondria functions. Based on the unsupervised cluster results, we calculated the average expression level of each gene in the stressed group (*n* = 10) and in the non-stressed control group (*n* = 10). The microarray data of 270,000 spots across all 60 microarray chips used for 20 RNA samples were filtered by uniform statistic and bioinformatic criteria (7), which generated 85 genes with informative expression profiles.

**Figure 2:**
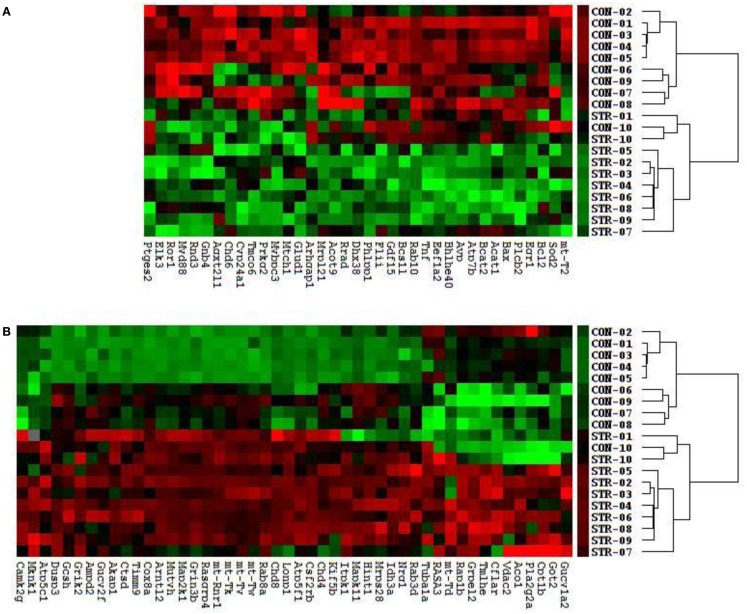
**Clustering analysis of 48 upregulated and 37 downregulated genes across all 20 amygdala samples generates double dendrograms and heat maps that distinguish the stressed amygdala specimens from the controls (A,B)**. The identification of these up- and downregulated genes provides candidates for network analysis. The ratios and *p*-values (*t*-test) were calculated between the stressed (*n* = 10) and control specimens (*n* = 10), resulting in the identification of 85 genes (*p* < 0.01) and among these 55 altered genes (*p* < 0.01) were further selected using a high stringency algorithm that includes fold change and fault discovery rate in the stressed amygdala as compared to the controls (Tables 1 and 2).

We identified a cluster and heatmap of 85 genes with significantly expressed RNA derived from the amygdala complex of the 10 control rats and the 10 stressed rats (Figure 2). The resultant dendrograms for all of these 85 genes and 20 amygdala complex specimens are classified as control group from CON-01 to CON-10 and stressed group from STR-1 to STR-10 in Figure 2; these analyses separated the stressed amygdala from those of the controls by their gene expression profiles. Figure 2A reveals 37 downregulated gene profiles and Figure 2B reveals 48 upregulated gene profiles.

### Pathway analysis of up- and downregulated genes

Pathways containing protein products of the up- and downregulated genes were determined using the Ingenuity Pathway Analysis (IPA) program. Table 1 illustrates the 34 upregulated genes and 21 downregulated genes in the amygdala after stress. A high stringency algorithm was used, including fold changes and FDR comparing stressed to controls (Tables 1 and 2). The tables include gene symbols, full names of genes listed in Genecards, fold change [ratio of stress and control, log(2) of the ratio], *p*-value, and fault discover rate (FDR).

**Table 1:** **Downregulated genes in amygdala (14 days after stress)**.

No	Gene symbols	Names of genes listed in Genecards	Fold change		Significance	
			Stress/CON	Log(2)	*p*	FDR
1	Eef1a2	Eukaryotic translation elongation factor 1 alpha 2	0.22	−2.20	0.0007	0.0044
2	Atp7b	ATPase, Cu++ transporting, beta polypeptide	0.31	−1.69	0.0062	0.0129
3	mt−Tl2	Mitochondrially encoded tRNA leucine 2 (CUN)	0.31	−1.68	0.0035	0.0088
4	Bhlhe40	Basic helix-loop-helix family, member e40	0.34	−1.58	0.0006	0.0044
5	Mtch1	Mitochondrial carrier 1	0.35	−1.53	0.0012	0.0047
6	Cyp24a1	Cytochrome P450, family 24, subfamily A, polypeptide 1	0.35	−1.52	0.0145	0.0199
7	Bcl-2	B-cell CLL/lymphoma 2	0.37	−1.42	0.0000	0.0014
8	Bcs1l	Mitochondrial chaperone BCS1	0.41	−1.30	0.0079	0.0134
9	Tnf	Tumor necrosis factor-alpha	0.42	−1.25	0.0005	0.0044
10	Acot9	Acyl-CoA thioesterase 9	0.44	−1.20	0.0024	0.0071
11	Rnd3	RND3 Rho family GTPase 3	0.44	−1.20	0.0056	0.0122
12	Dhx38	DEAH (Asp–Glu–Ala–His) box polypeptide 38	0.46	−1.13	0.0075	0.0133
13	Bcat2	Branched chain amino-acid transaminase 2, mitochondrial	0.47	−1.09	0.0097	0.0155
14	GDF-15	Growth differentiation factor 15	0.47	−1.08	0.0039	0.0091
15	Flii	Flightless I homolog	0.48	−1.06	0.0039	0.0091
16	Gnb4	Guanine nucleotide binding protein (G-protein), beta polypeptide 4	0.48	−1.05	0.0069	0.0133
17	Rrad	Ras-related associated with diabetes	0.49	−1.04	0.0041	0.0091
18	Acat1	Acetyl-CoA acetyltransferase 1	0.49	−1.03	0.0146	0.0199
19	Elk3	ETS-domain protein (SRF accessory protein 2)	0.50	−0.99	0.0073	0.0133
20	Phlpp1	PH domain and leucine rich repeat protein phosphatase 1	0.51	−0.97	0.0012	0.0047
21	Plcb2	Phospholipase C, beta 2	0.54	−0.90	0.0103	0.0159

*Approved symbol from the HUGO Gene Nomenclature Committee (HGNC) database*.

**Table 2:** **Upregulated genes in amygdala (14 days after stress)**.

No	Gene	Names of genes listed in Genecards	Fold change		Significance	
			Stress/CON	Log(2)	*p*	FDR
1	RASA3	Ras GTPase-activating protein 3	2.02	1.01	0.0040	0.0091
2	Dusp3	Dual specificity protein phosphatase 3	2.03	1.02	0.0107	0.0163
3	Gucy1a2	Guanylate cyclase 1, soluble, alpha 2	2.05	1.03	0.0023	0.0071
4	Grik2	Glutamate receptor, ionotropic, kainate 2	2.06	1.04	0.0012	0.0047
5	Mrps28	Mitochondrial ribosomal protein S28	2.21	1.14	0.0143	0.0199
6	Got2	Glutamic-oxaloacetic transaminase 2	2.27	1.18	0.0077	0.0133
7	Gucy2f	Guanylate cyclase 2F, retinal	2.34	1.23	0.0075	0.0133
8	Atp5c1	ATP synthase, H + transporting, mitochondrial F1 complex, gamma polypeptide 11	2.36	1.24	0.0111	0.0165
9	Akap1	A-kinase anchor protein 1	2.39	1.25	0.0098	0.0155
10	Idh3a	Isocitrate dehydrogenase 3 (NAD+) alpha	2.57	1.36	0.0034	0.0088
11	Cox8a	Cytochrome *c* oxidase subunit VIIIA (ubiquitous)	2.61	1.38	0.0024	0.0071
12	Cpt1b	Carnitine palmitoyltransferase 1B (muscle)	2.80	1.49	0.0074	0.0133
13	Atp5f1	ATP synthase, H+ transporting, mitochondrial Fo complex, subunit B1	3.02	1.59	0.0002	0.0029
14	mt-Tw	Mitochondrially encoded tRNA tryptophan	3.18	1.67	0.0011	0.0047
15	Kif5b	Kinesin family member 5B	3.21	1.68	0.0147	0.0199
16	CTSD	Cathepsin D	3.30	1.72	0.0017	0.0055
17	Nrg1	Neuregulin 1	3.34	1.74	0.0006	0.0044
18	mt-Ty	Mitochondrially encoded tRNA tyrosine	3.44	1.78	0.0011	0.0047
19	Timm9	Translocase of inner mitochondrial membrane 9 homolog	3.49	1.80	0.0011	0.0047
20	Rab8a	Member RAS oncogene family	3.54	1.82	0.0000	0.0014
21	Rasgrp4	RAS guanyl releasing protein 4	3.62	1.86	0.0008	0.0047
22	mt-Tk	Mitochondrially encoded tRNA lysine	3.69	1.88	0.0006	0.0044
23	mt-Rnr1	Mitochondrially encoded 12S RNA1	4.05	2.02	0.0001	0.0024
24	Camk2g	Calcium/calmodulin-dependent protein kinase II gamma	4.13	2.05	0.0032	0.0086
25	Grin3b	Glutamate receptor, ionotropic, *N*-methyl-d-aspartate 3B protein	4.77	2.25	0.0016	0.0055
26	Itpk1	Inositol 1,3,4-trisphosphate 5/6 kinase2	5.59	2.48	0.0006	0.0044
27	Csf2rb	Colony stimulating factor 2 receptor, beta, low-affinity (granulocyte-macrophage)	6.11	2.61	0.0005	0.0044
28	Tmlhe	Trimethyllysine hydroxylase, epsilon	6.71	2.75	0.0007	0.0045
29	Arntl2	Aryl hydrocarbon receptor nuclear translocator-like 2	7.86	2.97	0.0001	0.0028
30	Mutyh	mutY homolog (*E. coli*)	7.92	2.99	0.0141	0.0199
31	Rap1b	Ras family small GTP binding protein RAP1B2	10.13	3.34	0.0014	0.0053
32	Lonp1	Lon peptidase 1, mitochondrial	12.04	3.59	0.0016	0.0055
33	Grpel2	GrpE-like 2, mitochondrial (*E. coli*)	13.49	3.75	0.0009	0.0047
34	mt-Td	Mitochondrially encoded tRNA aspartic acid	14.17	3.82	0.0032	0.0086

Molecular networks associated with mitochondrial functions were constructed based on up- and downregulated genes from the amygdala complex associated with exaggerated fear (13). Network analysis indicated that four reregulated molecular networks were associated with metabolic molecules and neurotransmitter systems, as well as with translational factors in the amygdala complex (Figure 2). Mitochondrial dysfunction involved in endocrine and neuronal signaling was evident in the amygdala. Detailed molecular correlations of these reregulated molecular pathways and the symptoms of PTSD such as exaggerated fear are noted (Figure 2). The results also suggest possible molecular targets for pharmacological intervention following exposure to traumatic stress.

In the network analysis, the networks identified are presented as graphs indicating the molecular relationships between gene products. Gene products are represented as nodes, and a biological relationship between two nodes is indicated by a line. The intensity of the node color indicates the degree of upregulation (red) or downregulation (green). Gene products in uncolored nodes were not identified as differentially expressed in our experiment, but were integrated into the computationally generated networks on the basis of the evidence stored in the IPA knowledge base indicating a relevance to this network.

**Figure 3:**
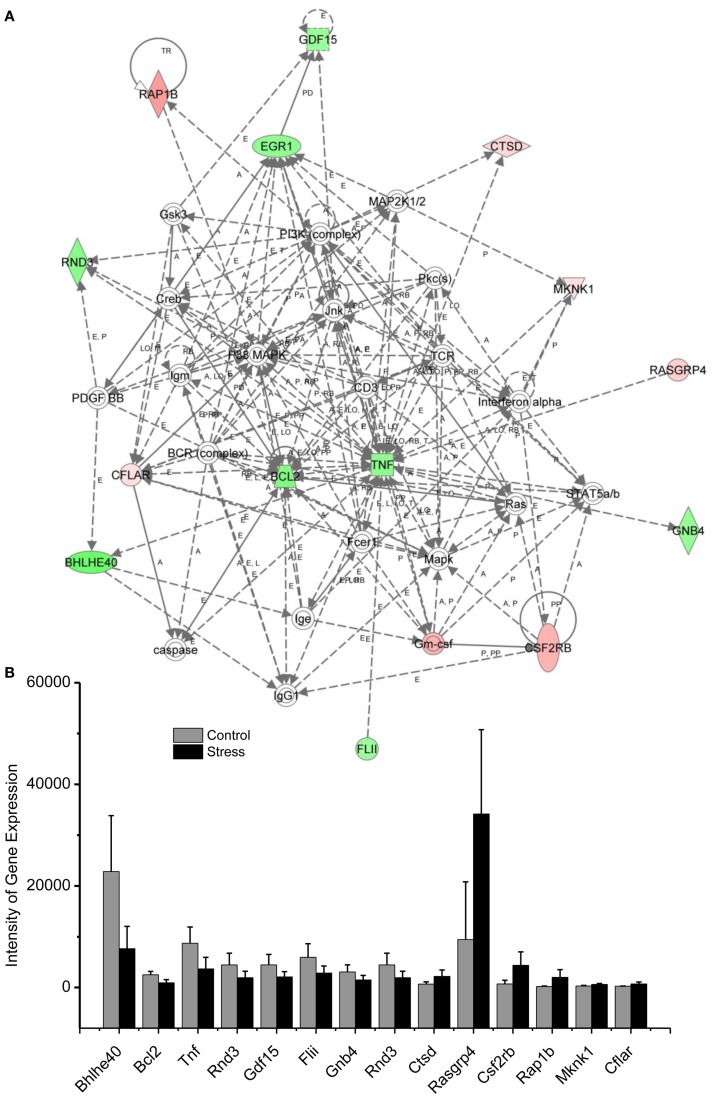
**In (A), IPA associates stress reregulated mitochondrial- related genes with the network containing Bcl-2 and TNF are shown**. **(B)** is a bar graph plotted as mean ± SD of the intensity of up- or downregulated genes in the stressed (*n* = 10) and control amygdala (*p* < 0.01, *n* = 10) for the network associated with Bcl-2 and TNF in the amygdala 14 days after stress. The order is plotted according to fold change, from downregulated with lowest negative value to upregulated with highest positive value.

The node shapes denote enzymes, phosphates, transmembrane receptors, cytokines, channels, transcription factors, G-protein-coupled receptors, growth factors, or nuclear receptor, as shown in Figure 3. Canonical pathway analysis identified the canonical pathways from the IPA library that were most significantly related to the input data set. The significance of the association between the data set and the canonical pathway was determined based on two parameters: (1) a ratio of the number of genes from the data set that map to the pathway divided by the total number of genes that map to the canonical pathway and (2) a *p*-value calculated using Fischer’s exact test determining the probability that the association between the genes in the data set, and the canonical pathway is due to chance alone.

**Figure 4:**
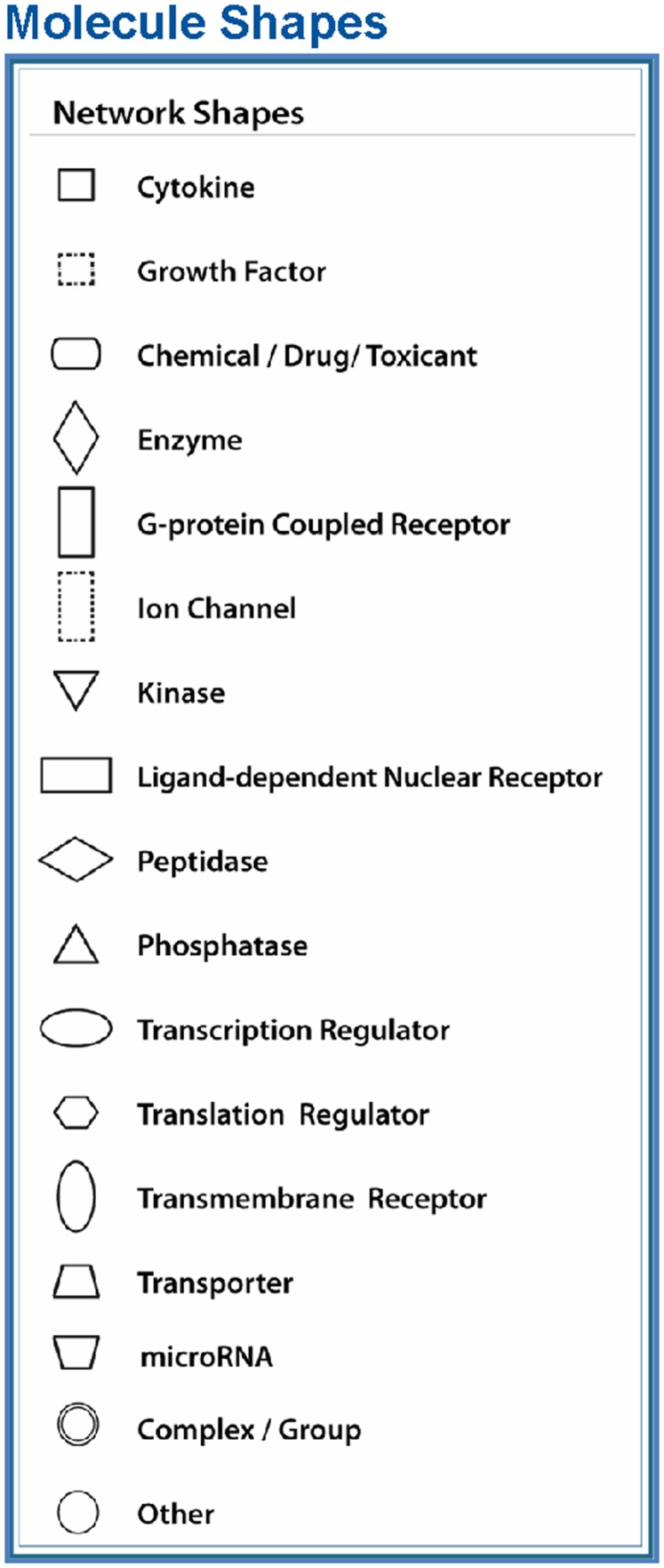
**Node (gene) and edge (gene relationship) symbols are described in the figure**. The intensity of the node color-(*red*) indicates the degree of upregulation. Genes in uncolored nodes are not identified as differentially expressed genes in our experiment and are integrated into the computationally generated networks on the basis of the evidence stored in the IPA knowledge memory indicating a relevance to this network. The node shapes denote enzymes, phosphatases, kinases, peptidases, G-protein-coupled receptors, transmembrane receptors, cytokines, growth factors, ion channels, transporters, translation factors, nuclear receptors, and transcription factors.

**Figure 5:**
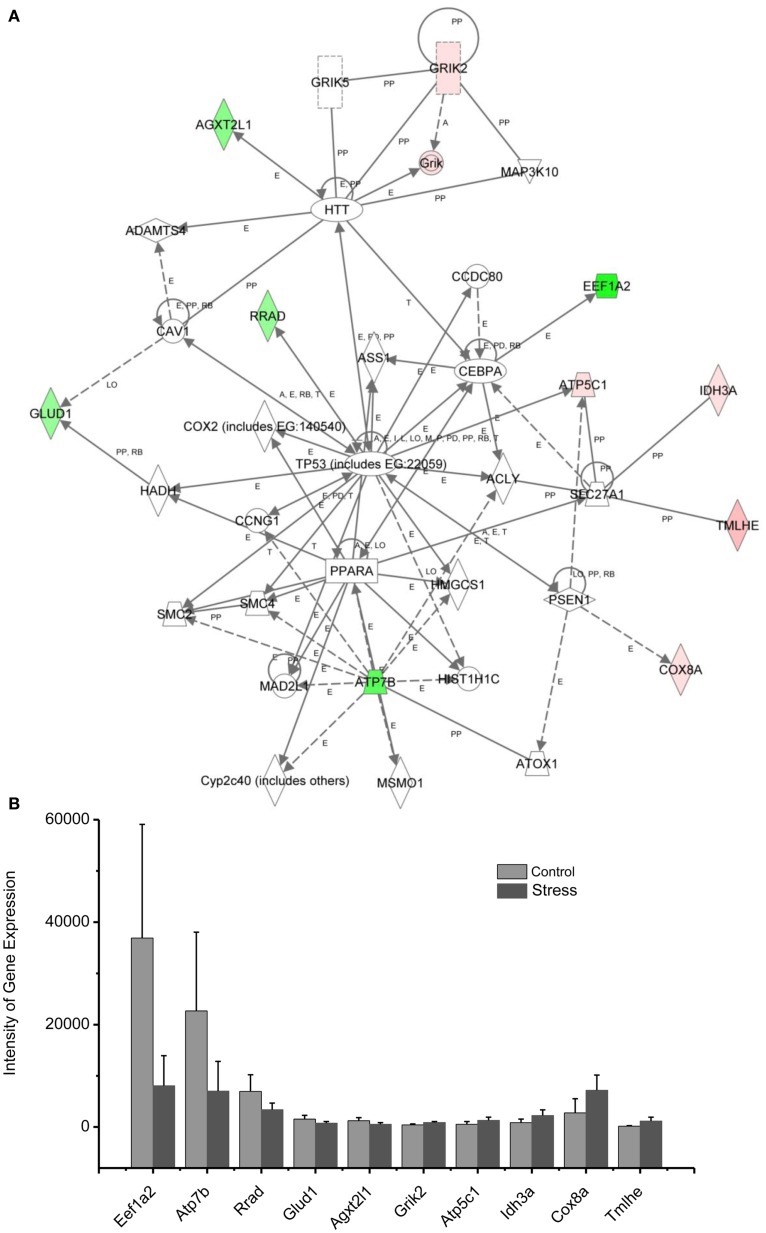
**In (A), IPA associates stress up- or downregulated mitochondrial-related genes with the network containing TP53, PPARA, and HTT**. **(B)** is a bar graph plotted with mean ± SD of the intensity of up- or downregulated genes for the stressed (*n* = 10) and control amygdala (*p* < 0.01, *n* = 10) in the network associated with TP53, PPARA, and HTT in the amygdala 14 days after stress. The order is plotted according to fold change, from downregulated with lowest negative value to upregulated with highest positive value.

**Figure 6:**
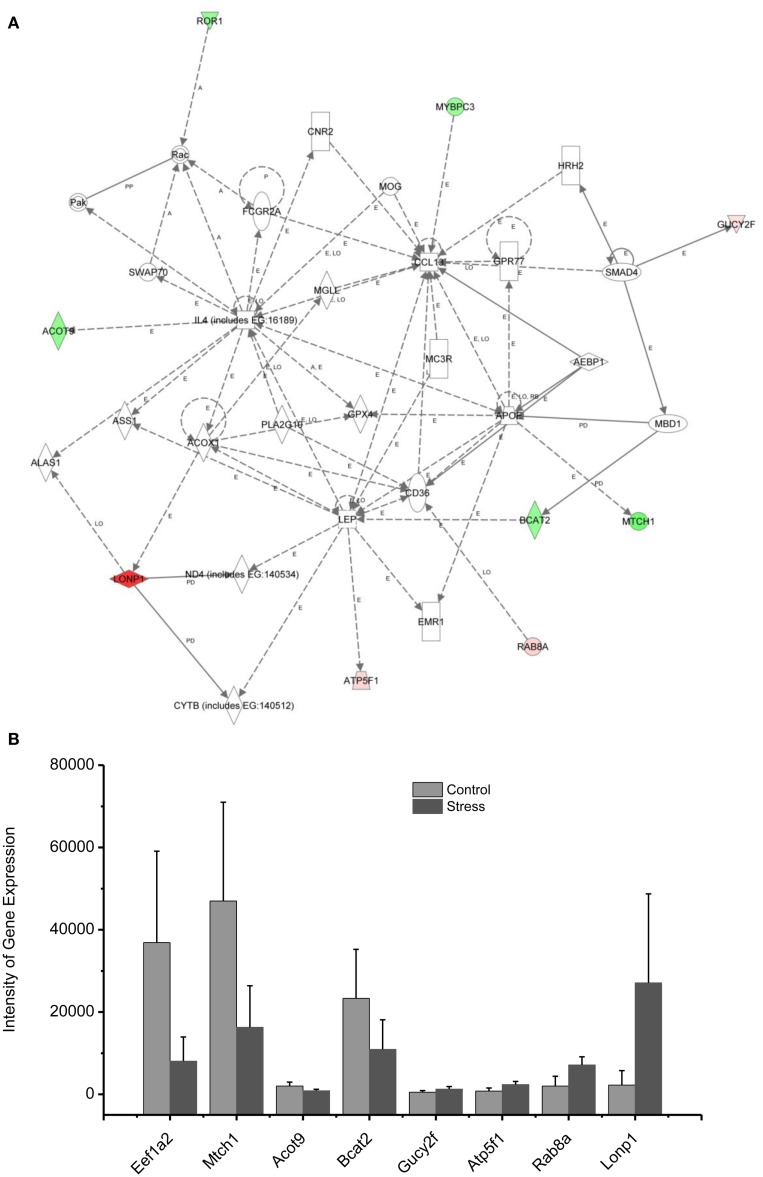
**In (A), IPA associates stress up- or downregulated mitochondrial-related genes with the network containing LEP and CL1**. **(B)** is a bar graph plotted with mean ± SD of the intensity of up- or downregulated genes for the stressed (*n* = 10) and controlled amygdala (*p* < 0.01, *n* = 10) in the network associated with LEP and CL1 in the amygdala 14 days after stress. The order is plotted according to fold change, from downregulated with lowest negative value to upregulated with highest positive value.

**Figure 7:**
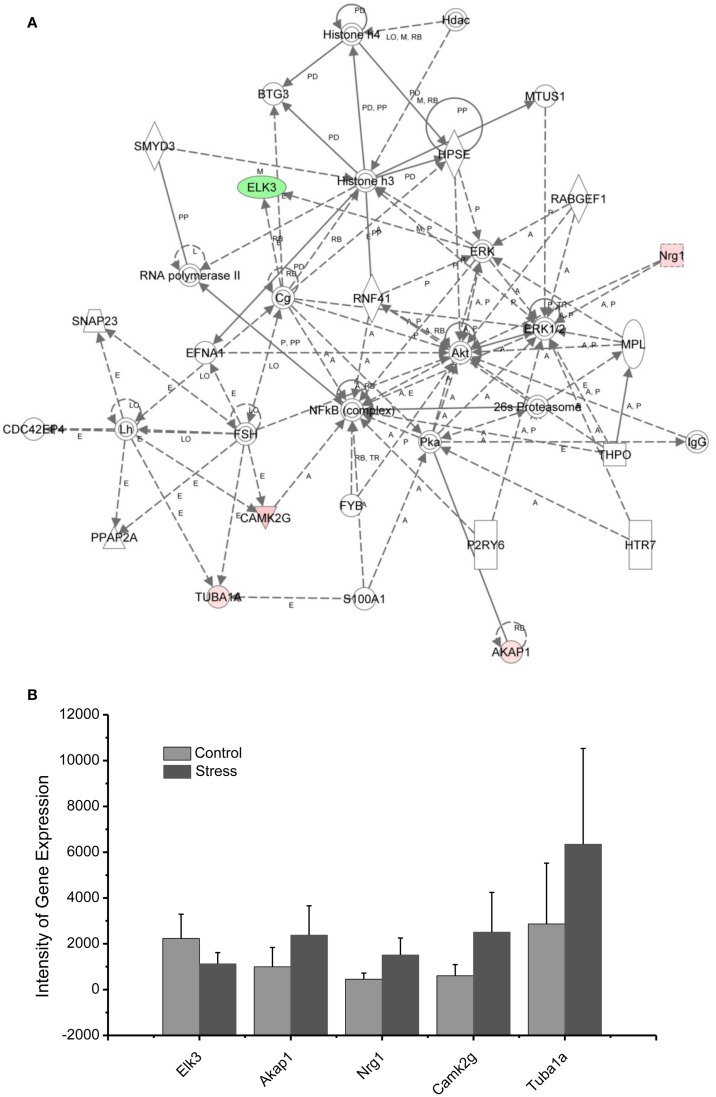
**In (A), IPA associates stress reregulated mitochondrial- related genes with the network containing NFkb, Akt, and Histone 3**. **(B)** is a bar graph plotted with mean ± SD of the intensity of up- or downregulated genes for the stressed (*n* = 10) and controlled amygdala (*p* < 0.01, *n* = 10) in the network associated with NFkb, AKt, and Histone 3 in the amygdala 14 days after stress. The order is plotted according to fold change, from downregulated with lowest negative value to upregulated with highest positive value.

To reveal specific and detailed interactions among up- and downregulated genes in the amygdala, and to further identify gene targets for pharmacological agents, four molecular networks were delineated: (1) Bcl-2 and TNF centered inflammatory and apoptotic signaling networks as shown in Figure 4; (2) energy production and neurotransmitter-mediated signaling pathways as shown in Figure 5; (3) transcriptional factor and chromatin remodeling pathways as shown in Figure 6; (4) immune mediator-related pathways as shown in Figure 7. The genes in the pathways were selected based on recent literature in the IPA database and relevance to neurological dysfunction, psychiatric disorders, and exaggerated fear response associated with amygdala functions as addressed in the Section “Discussion.” Within these networks, 34 (62%) of the genes were significantly upregulated (Table 2; Figure 2B) and 21 (38%) of the genes were significantly downregulated (Table 1; Figure 2A). In the network analysis, these genes display particular relevance to the symptoms of PTSD.

### Validation of the Rap1b and Arhgap1 gene expressions in the amygdala (microarray) and blood (qRT-PCR)

Quantitative real-time PCR (qRT-PCR) was used to validate and to confirm gene expression results obtained from microarray analysis in our previous published results (4). The relative mRNA levels of identified genes measure by qRT-PCR were in agreement with the data detected by microarray from 92 to 67% on five genes examined (4). In an attempt to correlate biomarkers in peripheral tissue (blood) with the profile of dysregulated genes in the amygdala, Rap1b and Arhgap1 were chosen in the current study because of upregulation of (Rap1b) and downregulation of (Arhgap1) expressions observed in stressed amygdala and blood as well as because Rap1b is of known relevance to abnormal fear response (21). The results show that the relative mRNA levels of these genes measured by qRT-PCR (blood, *n* = 8, *p* < 0.01) were in agreement with the data detected by microarray (amygdala, *n* = 10, *p* < 0.01) experiments (shown in Figures 1A,B in Supplementary Material).

## Discussion

Animal models of PTSD offer opportunities to identify mechanisms, potential biomarkers, and treatment targets for this disorder. In this study, we have shown that a substantial number of up- and downregulated genes related to mitochondrial function in the amygdala are associated with an animal model of PTSD based on exaggerated startle and retardation of growth (9, 13). These mitochondrial-function genes have been previously related to neurological dysfunction, psychiatric disorders, and stress response (4, 6). In addition, we have identified novel mitochondrial-function-related genes associated with endocrine and neuronal signaling (4, 5, 22).

Traumatic stress up- or downregulation of anti-inflammatory and anti-apoptotic cellular pathways are evidenced in the amygdala 14 days after stress, as indicated in Figure 4 and Tables 1 and 2. Seven genes, including RAP1B, Cathepsin D (CTSD), MKNK1, RASGRP, CSF2RB, GMCSF, and CFLAR are upregulated and nine genes, including tumor necrosis factor (TNF), Bcl-2, GDF-15, early growth response (Egr1), GNB, FLII, BHLHE40, RND3, and GNB4 are downregulated in the inflammatory and apoptotic signaling networks in the amygdala.

Levels of inflammatory cytokines in blood samples have been used as biomarkers of PTSD vulnerability and resilience in traumatized individuals (23–26). TNF-α and related anti-inflammatory cytokines in the peripheral blood have been correlated with symptoms of PTSD, including re-experiencing, avoidance, and hyperarousal, and with PTSD total symptom score (23, 24, 26). In a single stress rat model, increased neuronal apoptosis has been observed in the medial prefrontal cortex (mPFC) 7 days after stress exposure indicating that apoptotic-related molecules are sensitive to traumatic stress (27). Emerging data indicate that TNF and the anti-apoptotic protein Bcl-2 exert a neuroprotective effect in the brain against traumatic stress (28–30).

Using network analysis, current results reveal for the first time that the 15 genes of the inflammatory pathway in the amygdala complex are up- or downregulated in the exaggerated fear response (Figure 4). These inflammatory signaling molecules may participate in the evolution of PTSD to a chronic disorder because they remain up- or downregulated 14 days after the stress protocol. Bcl-2, an integral outer mitochondrial membrane protein, was initially found to block the apoptotic death of lymphocytes, and over expression of Bcl-2 in transgenic mice reduced the fear response (31, 32). In the current stress model of PTSD, the expression of Bcl-2 is downregulated in the amygdala, indicating that both neuronal inflammatory and cellular apoptosis signaling pathways contribute to the exaggeration of fear following traumatic stress. The network analysis encourages future studies to detail the cellular mechanisms that are evidenced to underlie PTSD (4, 5, 27, 33). For example, the Bcl-2 mediated apoptotic cellular process may contribute not only to the degenerated amygdala structure in PTSD patients but also to the impairment of the controlling function of the amygdala in the fear response of PTSD patients. This process may also be observable in animal models of PTSD (27, 34–36).

Among the downregulated genes, the basic helix–loop–helix transcription genes, BHLHE, have been associated with symptoms of PTSD such as sleep disturbance. For example, BHLHE40 and BHLHE41 are mainly expressed in the suprachiasmatic nucleus of the hypothalamus, which governs circadian rhythm (37). BHLHE40 synchronizes the circadian rhythm at the intracellular level with the external light–dark cycle. The current study demonstrates for the first time that downregulated BHLHE40 expression in the amygdala is associated with exaggerated fear following traumatic stress. Thus BHLHE40 may be a target for treatment of the disruption of sleep associated with PTSD, a symptom that is often resistant to present pharmacotherapy. Changes in the light/dark cycle have also been shown to disrupt memory of conditioned fear (38–40). Thus, the circadian system may relate to anxiety and altered cognition following traumatic stress.

In addition to the Hub molecules such as Bcl-2 and TNF in the inflammatory and apoptotic pathways, other up- or downregulated gene products in the pathways could contribute to the exaggerated fear response. Although little has been published associating these molecules with exaggerated fear or other PTSD symptoms, they have been related to neuronal regulation. For example, GDF-15 is a potent trophic factor in the recovery of 6-OHDA-lesioned midbrain dopaminergic neurons (41). Downregulation of GDF-15 may jeopardize neuronal survival in the amygdala following traumatic stress. Mitochondrial carrier homolog 1 (Mtch1) is an outer mitochondrial membrane protein and overexpression of Mtch1 causes mitochondrial depolarization and apoptosis, likely via the permeability transition pore (42). Egr transcriptional regulators such as Egr1 have been widely recognized as molecules essential for emotional learning and memory (43, 44). Moreover, Egr1-deficient mice have impairments in late phase hippocampal LTP and consolidation of some forms of amygdala-dependent memory through a protein phosphatase calcineurin-mediated mechanism (43, 44). Thus, downregulated Egr1 following traumatic stress may impair cellular processes associated with emotional learning.

### Traumatic stress up- and downregulated genes in the amygdala associated with suicide

Post-traumatic stress disorder significantly increases risk for suicide, even after adjusting for depression, which is often comorbid. Therefore, it appears that traumatic stress exposure and its consequences are risk factors for suicide in some people (45, 46). Correlation of mechanisms underlying suicide anxiety can help in understanding the cellular and molecular connections between these two mental disorders. Using molecular network analysis of genes up- or downregulated by traumatic stress, as shown in Figure 2 and Tables 1 and 2, genes including TUBA1, CTSD, and Grik2 have been associated with suicidal events (47, 48). Dysregulation of each of these genes produces profound damage to the brain.

Mutations of TUBA1A in this gene cause lissencephaly type 3 (LIS3), a neurological condition characterized by microcephaly, mental retardation, early-onset epilepsy, and defective neuronal migration (49). TUBA1 is also an interacting cytoskeleton protein that has a functional connection to glutamate, GABA, and serotonin receptors (48). Mutations in the CTSD gene are involved in the pathogenesis of AD and Niemann–Pick disease (50–53).

A recent proteomic study on post mortem brains of suicide victims shows that both TUBA1A and CTSD are highly expressed in the prefrontal cortex and amygdala of suicide victims, suggesting that these genes may be a risk biomarker for suicide (48). As shown in Figure 4, S100 Calcium Binding Protein A1 (S100A1; one of the proteins in the super family of S100, also called P11) interacts with TUBA1A and may regulate the function of TUBA1 under stressful conditions (54). In fact, in a recent clinical study, the mRNA level of S100A10 was found to be a potential adjunctive biomarker for the assessment of suicide risk in anxiety disorders (55).

Kainate receptors are members of the ionotropic class of glutamate receptors and are highly expressed in the amygdala neuronal circuitry (56–60). These kainate receptors have been identified both pre- and post-synaptically. They modulate inhibitory and excitatory postsynaptic currents in the basolateral amygdala circuitry and play a role in synaptic plasticity associated with emotional learning and memory (56–60). Thus upregulated Grik2 and Grik genes, as found in the current study, may participate in the emotional hyperarousal following traumatic stress. In addition, recent pharmacogenomic studies of antidepressant treatment-emergent suicidal events report associations with polymorphisms of Grik2 receptor genes in depressed patients (47). The network analysis from the present results suggests that the pharmacologic agents that target specific subtypes of kainate receptors may be therapeutic medications for the treatment of PTSD with suicidal ideation.

In summary, network analysis reveals that signaling pathways associated with TUBA1A, CTSD, and Grik2 are potential biomarkers and therapeutic targets for PTSD with suicidal ideation.

### Up/downregulation of genes in the amygdala subserving energy production and neurotransmitter synthesis

Diminished interest and participation in activities along with detachment and lack of positive emotions may be seen in PTSD as energy loss or mental fatigue. This decreased energy is a common complaint of service members after repetitive traumatic stress, including PTSD and TBI (61). While the mechanisms underlying the mental fatigue are speculated to be due to a shortage of energetic molecules in the brain, the detailed mechanism remains elusive. The relationship between mitochondrial gene expression in the amygdala and exaggerated fear provides an approach to the cellular and molecular basis of mental fatigue after trauma. As shown in Figure 5, two citrate cycle genes, Idh3a and Cpt1b, were upregulated. Idh3a promotes ATP production by catalyzing the oxidative decarboxylation of isocitrate to 2-oxoglutarate. Cpt1b generates ATP from lipids via beta-oxidation of fatty acids (62–64). Expression of ATP5C1 is enhanced. ATP5C1 is the mitochondrial membrane ATP synthase. Suppression of the ATP5C1 gene caused a decrease in cell proliferation and ATP production (65). Enhanced expression of ATP5C1 may suggest an enhanced demand for energy in the amygdala in response to traumatic stress associated with exaggerated fear. Indeed, enhanced expression of ATP5C1 was also found in the prefrontal cortex of PTSD postmortem brains, indicating that the ATP5C1 gene could be a biomarker for the diagnosis of PTSD (4).

Mitochondrial genes supporting biosynthesis are differentially regulated under conditions of stress. Got2, which is involved in arginine, tyrosine, and tryptophan biosynthesis, is upregulated; Acot1 and Bcat2, two enzymes involved in fatty acid metabolism, as well as synthesis of valine, leucine, and isoleucine, and degradation of lysine, are downregulated. Tryptophan is a precursor of serotonin, which is thought to contribute to emotional well-being and is related to depression, which is highly comorbid with PTSD (66, 67). In addition, selective serotonin reuptake inhibitors (SSRIs) are a first line treatment for PTSD.

These results indicate that altered regulation of expression of genes involved with energy production and biosynthesis in the amygdala may be associated with the diminished interest, overall mental fatigue, and comorbid symptoms of depression in PTSD.

### Up/downregulation of genes in the amygdala associated with immune mediators and energy expenditure

Proinflammatory chemokines have been suggested to be involved in chronic stress, major depressive disorder, panic disorder, and PTSD (68–73). The chemokines IL4 and CCL13 are interconnected with the up- and downregulated genes shown in the network 3, including Lonp1, ATP5F1, Rab8A, and Gucy2F. Levels of chemokines including IL4, IL2, and TNF alpha in the plasma have been found to be significantly altered in some patients suffering from traumatic stress, including those with PTSD and those suffering from child abuse (74). CCL13 is a chemotactic cytokine that regulates leukocyte migration through interactions with G-protein-coupled receptors (75, 76).

The up- and downregulated genes shown in the network (Figure 6) are connected with Leptin (LEP), which is a key mediator in the regulation of food intake, body weight, and possibly memory. Dysregulation of LEP could be involved in the weight loss resulting from chronic and repetitive stress in our rat model of PTSD (11, 13, 77). Weight loss is a common symptom of depression, highly comorbid with PTSD. Indeed, elevated serum LEP levels were found in PTSD patients suffering from depressive symptoms caused by earthquakes and myocardial infarctions (78, 79). In addition, intraperitoneal injection of LEP mitigated the impairment of fear memory in the mouse deprived of rapid eye movement sleep (80). Thus, it appears that the elevation of LEP in patients with PTSD may be associated with elevated fear, similar to an observation made in the current rat model of PTSD.

### Up/downregulation of genes in the amygdala associated transcription factors and chromatin remodeling

As indicated in the signaling pathway shown in Figure 7, four genes, including neuregulin 1 (Nrg1), calmodulin-dependent kinases 2 gamma (Camk2g), tubuline (Tuba1A), and the A-kinase anchor protein 1 (AKAP1), are upregulated in the amygdala 14 days after stress, and E-twenty six (ETS) domain-containing transcription factor (ELK3) is downregulated. In addition to the functions of the individual up- or downregulated genes in Figure 7, these genes are interconnected with histone 3 and histone 4. Histone H3 and histone H4 are two of the five main histone proteins involved in the structure of chromatin in eukaryotic cells. Thus, the up- or downregulated genes in the network appear to affect transcription and chromatin remodeling.

Regulation of chromatin structure through post-translational modification of histone proteins is a critical step in the formation of long-term memory (81). As revealed in the network analysis, traumatic and repetitive stress leads to altered regulation of chromatin-related genes in the amygdala, including the upregulation of Nrg1, CamK2g, Tuba1A, and AKAP1, as well as the downregulation of ELK3 interacting with NF-kappa B, histone 3, and histone 4. Histone 3 and histone 4 drive epigenetic modifications and conformational changes in chromatin, stimulating the expression of neuroplasticity-related genes involved in traumatic memory and fear learning (82, 83). Histone acetylation mediated by BDNF gene transcription is involved in the consolidation of fear memory associated with an animal model of PTSD (84). This modulation could promote chromatin remodeling in the amygdala induced by glucocorticoids following traumatic stress. Indeed, activation of the glucocorticoid receptor (GR) under stress has also been shown to modify chromatin structure by post-translational modifications of histones and is considered to be a new therapeutic target for PTSD (85, 86).

Nuclear factor kappa-light-chain-enhancer of activated B cells (NF-κB), a protein complex that controls the transcription of DNA, interacts with up- or downregulated genes in network 4. Inhibition of NF-κB in the basolateral amygdala impairs the memory reconsolidation associated with auditory fear conditioning (87).

### Summary

The current study reveals that multiple signaling networks are up- or downregulated in the amygdala circuitry 14 days after traumatic stress. Traumatic stress significantly alters at least 55 genes associated with at least four pathways related to mitochondria functions in the stressed amygdala. These molecules include well-studied and -documented networks as well as novel networks that have not been well documented in previous literature. These findings provide a guide for further studies associated with diagnostic biomarkers, therapeutic molecular targets, and identification of optimal strategies for fostering resilience before and after traumatic stress. In addition, current analyses suggest new targets for pharmacological intervention to treat PTSD and suicide. Suicide-related genes revealed in the stressed amygdala indicate that dysregulated signaling pathways interweave suicide and fear. These results elucidate the molecular pathways underlying PTSD as a road to diagnosis and treatment.

## Conflict of Interest Statement

The Guest Associate Editor Yumin Zhang declares that, despite being affiliated to the same institution as authors He Li, Stanley E. Smerin, Lei Zhang, Min Jia, Guoqiang Xing, Jillian Wen, David Benedek, and Robert Ursano, the review process was handled objectively and no conflict of interest exists. The authors declare that the research was conducted in the absence of any commercial or financial relationships that could be construed as a potential conflict of interest.

## Supplementary Material

The Supplementary Material for this article can be found online at http://www.frontiersin.org/Journal/10.3389/fneur.2014.00164/abstract

## Footnotes

^1^http://rana.lbl.gov/EisenSoftware.htm

^2^www.ncbi.nlm.nih.gov/gene

^3^http://david.abcc.ncifcrf.gov
