# Supplementary material for: Mitochondrial Gene Expression Profiles and Metabolic Pathways in the Amygdala Associated with Exaggerated Fear in an Animal Model of PTSD
Source: Front Neurol. 2014 Sep 23;5:164. doi: 10.3389/fneur.2014.00164 (PMC4172054; doi:10.3389/fneur.2014.00164)
Supplement: Supplementary file 1 [file DataSheet_1.DOC]

**Supplementary Supporting Materials**

Methods for supplementary experiments:

Rat blood collection and real time PCR

### All rats were handled and weighed before each study began. Animals either remained undisturbed in their home cages as controls or were exposed to inescapable tail shock protocol and 3 ml rat whole blood was collected using PAXgene Blood RNA Tube (PreAnalytix, Qiagen). The blood samples were incubated at room temperature for 1 hour and then stored and transferred at -20ºC according to the manufacture’s instruction. RNA was extracted using the RNA extraction kits (Qiagen). The extracted RNA was then used for reverse transcription. Reverse Transcription was carried out with the SuperScript First-Strand Synthesis System ([Life Technologies](http://www.lifetechnologies.com/us/en/home/life-science/pcr/reverse-transcription/reverse-transcriptase-enzymes/superscript-iii-reverse-transcriptase.html), CA) for RT-PCR. cDNA was generated from 5 μg of total RNA for each sample using Superscript III RT (reverse transcriptase) and oligo (dT) primers ([Life Technologies](http://www.lifetechnologies.com/us/en/home/life-science/pcr/reverse-transcription/reverse-transcriptase-enzymes/superscript-iii-reverse-transcriptase.html), CA, USA) in 50 μl RT-PCR mix. Gene expression assays, including primers and probe, were ordered from Life Technologies. The assay number of Rn00821725_g1 is for the gene of Rap1b, Rn01484943_m1 for Arhgap 1 respectively. To exclude the differences in RNA-content resulted from differences in sample weights, the target genes were normalized to a house keeping gene of β-actin (Applied Biosystems, CA). All samples were assayed in duplicates, using the ABI 7900HT real-time PCR system. A total of 20 μl of final qPCR reaction volume was used, containing 1x qPCR reagent and 2 μl of the reverse transcription products. The relative concentration of the target genes was calculated using 2^-ΔΔCt^ method (Jiang et al., 2009;Su et al., 2008;Jia et al., 2012;Su et al., 2008).

**Supplementary Figure Legend**

Supplementary Fig. 1A. Expression profiles of the target genes measured by microarray in amygdala and qRT-PCR (Fig. 1B) in the blood.

The relative RNA expression levels of two targeted genes, Rap1b and Arhgap1, were determined by microarray (S.Figure 1A, n=10) and qRT-PCR (S.Figure 1B, n=8) techniques, respectively. The asterisks indicate statistic significance (*p*<0.01) between the control and stressed groups 14 days following the termination of stressed protocol.

Supplementary Reference List

Jia M, Meng F, Smerin SE, Xing G, Zhang L, Su DM, Benedek D, Ursano R, Su YA, Li H. Biomarkers in an Animal Model for Revealing Neural, Hematologic, and Behavioral Correlates of PTSD. J.Vis.Exp. 2012.

Jiang X, Xing G, Yang C, Verma A, Zhang L, Li H. Stress impairs 5-HT2A receptor-mediated serotonergic facilitation of GABA release in juvenile rat basolateral amygdala. Neuropsychopharmacology 2009; 34:410-423.

Su YA, Wu J, Zhang L, Zhang Q, Su DM, He P, Wang BD, Li H, Webster MJ, Rennert OM, Ursano RJ. Dysregulated mitochondrial genes and networks with drug targets in postmortem brain of patients with posttraumatic stress disorder (PTSD) revealed by human mitochondria-focused cDNA microarrays. Int.J.Biol.Sci. 2008; 4:223-235.

A

B
